# Biologic Evidence Required for Zika Disease Enhancement by Dengue Antibodies

**DOI:** 10.3201/eid2304.161879

**Published:** 2017-04

**Authors:** Scott B. Halstead

**Affiliations:** Uniformed Services University of the Health Sciences, Bethesda, Maryland, USA

**Keywords:** Zika, dengue, DENV, viruses, flaviviruses, infectious diseases, vector-borne infections, antibodies, disease enhancement, Zika virus

## Abstract

The sudden appearance of overt human Zika virus infections that cross the placenta to damage fetal tissues, target sexual organs, and are followed in some instances by Guillain-Barré syndrome raises questions regarding whether these outcomes are caused by genetic mutations or if prior infection by other flaviviruses affects disease outcome. Because dengue and Zika viruses co-circulate in the urban *Aedes aegypti* mosquito–human cycle, a logical question, as suggested by in vitro data, is whether dengue virus infections result in antibody-dependent enhancement of Zika virus infections. This review emphasizes the critical role for epidemiologic studies (retrospective and prospective) in combination with the studies to identify specific sites of Zika virus infection in humans that are needed to establish antibody-dependent enhancement as a possibility or a reality.

Recently, polyclonal and monoclonal dengue virus (DENV)–elicited antibodies have been shown to neutralize or enhance Zika virus infection in vitro (*1*,*2*). Three studies have shown that monoclonal antibodies to the DENV fusion loop epitope reliably enhanced Zika virus infection (i.e., antibody-dependent enhancement [ADE]) in Fc receptor–bearing K-562 human myelogenous leukemia cells or the U-937 human monocytic cell line (*1*–*3*). By contrast, broadly neutralizing DENV monoclonal antibodies directed at a conformational quaternary epitope on the virion formed at the interface of 2 envelope dimer epitopes (EDE1 and EDE2) potently neutralized Zika virus in a picomolar range similar to the neutralization of DENV (*2*). X-ray crystallographic structures of antigen binding fragments of EDE1 and EDE2 in complex with the Zika virus envelope protein have been obtained (*4*). These observations raise important questions about the past and future of human infections with Zika virus.

Outside Africa, Zika virus occupies the same epidemiologic niche as do the DENVs. During the ongoing pandemic in the Western Hemisphere, infections in the sequence of DENV followed by Zika virus must have occurred often and will continue to occur. Might placental transfer of Zika virus or Guillain-Barré syndrome (GBS), fueled by the ADE phenomenon, occur specifically in DENV-immune persons? If Zika virus infections are enhanced by DENV antibodies, might ADE also occur after administering a dengue vaccine? Indeed, if Zika virus infections can be enhanced by DENV antibodies, might that also be an outcome following administration of a poorly protective Zika virus vaccine? Correspondingly, might Zika virus antibodies enhance DENV infections? These crucial questions require biologically valid answers. What information do we need to answer them, and where shall we start looking?

Zika virus, a member of the *Flavivirus* genus, is maintained in complex African zoonotic cycles, spilling from time to time into the *Aedes aegypti* mosquito urban transmission cycle (*5*). This spillover might be a very old phenomenon. Because many flaviviruses infect humans in Africa, it was logical to ask if antibodies to these viruses enhanced Zika virus infections. The answer obtained in vitro was affirmative (*6*). After having been first isolated in Africa in 1947, human Zika virus disease remained sparse and mild, with no reports of diverse clinical syndromes associated with infection (*7*). Nor was Zika disease reported from India or Southeast Asia, where Zika virus and DENV co-circulated, evidenced by detection of neutralizing antibodies in humans as early as 1954 (*8*–*10*). Zika virus was isolated from *Ae. aegypti* mosquitoes collected in rural Malaysia in 1966 (*11*). No alarms were raised in Asia until 2007, on the Yap Islands in the Western Pacific, when it was estimated that ≈900 cases of a mild febrile exanthema caused by Zika virus infections had occurred among a total population of ≈7,000 (*12*). Then, during 2013–2014, on Tahiti, a Zika virus epidemic was followed in 4 weeks by an outbreak of GBS (*13*). Of the 42 case-patients with Zika virus infection and subsequent GBS, 95% had evidence of prior DENV infection, although this percentage did not differ from that of controls (*13*). This illness phenomenon spread to South America, where remarkably, GBS often followed acute Zika virus infections by only a few days (*14*–*17*). Then, abruptly, in Brazil, Zika virus was found to destroy fetal tissues (*18*). Next, it was learned that Zika virus infected the male reproductive tract and could be sexually transmitted (*19*).

Is the expanded pathogenicity of Zika virus the result of viral mutations, or might DENV ADE play a role, or are both true? An opinion has emerged that Zika virus genomes in Asia have acquired 1 or more mutations, contributing to its newly emerged pathogenicity (*5*,*20*). A recent analysis of full-length RNA sequences of 84 Zika virus genomes in Africa, Asia, the Pacific region, and the Americas has noted a stable amino acid change in the matrix protein of Asian viruses that accompanied placental invasiveness and GBS in Tahiti and the Americas (*21*). It is not clear what specific biologic properties might have been acquired by Zika virus that contributed to the observed new behaviors. The original strain of Zika virus recovered in Uganda in 1947 was neurotropic for 6-week-old mice (*22*). A strain genetically similar to the prototype in Africa productively infected human neural progenitor cells in vitro, dysregulating cell growth (*23*). In type 1 interferon receptor knockout mice or mice inoculated with type 1 interferon receptor–blocking antibodies, subcutaneous inoculation of a strain of Zika virus from French Polynesia infected maternal trophoblasts and resulted in apoptosis. In infected pregnant female mice, Zika virus crossed into the fetal circulation, where it infected endothelial cells, resulting in apoptosis and greatly impaired fetal circulation leading to ischemia and fetal loss (*24*).

Knowledge that a microbial disease is worsened by ADE derives from 2 evidentiary pillars: 1) epidemiologic evidence demonstrating that a unique syndrome or severe disease is significantly associated with persons who circulate antibodies (presumably “enhancing”) before infection, and 2) evidence of in situ replication by the causative organism in myeloid cells that serve as major targets of cellular infection. DENV disease enhancement was established by recording a strong association between severe disease in humans and a secondary-type DENV antibody response, by direct association of severe cases with sequential DENV infections in prospective cohort studies, and by the occurrence of severe disease during first DENV infections in infants born to dengue-immune mothers (*25*–*30*). This latter observation provides population-level evidence that DENV antibodies are the critical risk factor for the occurrence of severe DENV disease. However, severe disease in infants occurs only when infants acquire antibodies acquired by 2 or more lifetime DENV infections in the mother (*25*,*30*). Importantly, the same IgG antibodies that enhance DENV infections in infants are protective for the first several months after birth (*29*). Evidence that myeloid cells support intracellular DENV infection in vivo in humans derives from studies on tissues from virologically documented patients obtained during surgery, at autopsy, or by venesection (*31*–*34*). These studies, although few, are buttressed by the demonstration that DENV immune complex infection of Fc receptor–bearing cells leads directly to vascular permeability in mouse models (*32*,*35*–*40*).

To establish whether ADE caused by DENV antibodies modifies the course of Zika virus infections, evidence will be required from the same 2 pillars. The outcomes of DENV–Zika virus sequential infections might be diverse and complicated. First, antibodies derived from monotypic infections with each of the 4 DENV serotypes, if they enhance at all, might affect Zika virus infections differently. As an example, infections in the sequence DENV-1 followed by DENV-2 or DENV-3 then DENV-2 result in more severe clinical outcome than infections in any of the other 10 secondary infection sequences (*41*). Second, the interval between DENV followed by Zika virus infection might regulate disease severity. In dengue, the interval between initial and a heterotypic DENV infection exerts a remarkable bidirectional effect. Sequential DENV infections at a short interval (<2 years) blunt the clinical severity of responses to a second infection, whereas as the interval widens (2–20 years), the outcome becomes increasingly severe (*42*–*44*). Third, Zika virus infection enhancement could be critically dependent upon the parity of past DENV infections of the host. For example, as is true for dengue, a single prior DENV experience might enhance infection, whereas antibodies deriving from >2 past DENV infections might protect against further infection (*45*).

By using a simple protocol (Table), it should be possible to gather evidence using a case-control format comparing the frequency of a secondary flavivirus antibody response in convalescent-phase serum samples of persons experiencing any defined acute Zika virus disease syndromes, such as exanthematous fever, congenital Zika syndrome, or GBS (i.e., case-patients) with the prevalence of past DENV infection in Zika virus–infected age-, sex-, residence-, and ethnicity-matched persons from the same exposure group (i.e., controls). Such a comparison should be undertaken promptly in several different Zika virus–endemic locales. If these studies fail to provide evidence that prior flavivirus infection is a risk factor for any of the defined clinical outcomes of Zika virus infections, the search for Zika virus ADE can halt. However, if evidence compatible with ADE is obtained, it will be important to investigate the possibly complicated interactions between DENV and Zika virus infections alluded to here.

**Table:** Case–control epidemiologic research protocol for assessing DENV antibody enhancement of Zika virus syndromes*

Category	Description
Case-patients	Zika virus PCR positive or serologically positive symptomatic persons (i.e., with febrile illness, Guillain-Barré syndrome or congenital Zika syndrome) of any age who have convalescent-phase serum samples available to test for DENV IgG by ELISA.
Controls	Serum samples from age-, sex-, residence-, and ethnicity-matched controls with blood drawn at about the same time as each index case-patient. Ratio: 4 controls to 1 index case-patient.
Laboratory studies	1. Convalescent-phase serum samples from case-patients are tested by indirect pan-DENV IgG ELISA. (A positive result indicates that the Zika virus infection occurred in a DENV-immune person.)
2. Control serum samples are tested for Zika virus neutralizing antibodies.
3. Control serum samples are tested for past DENV infections by using indirect pan-DENV IgG ELISA.
4. DENV IgG ELISA–positive serum samples from case-patients and controls are tested for DENV serotypes 1–4 neutralizing antibodies.
5. Frequency of prior DENV infections in symptomatic Zika virus case-patients is compared with frequency of DENV antibodies in Zika virus–immune controls. A statistically significant increase in past DENV infection indicates enhancement; a statistically significant reduction indicates protection.
6. All comparisons should be made separately and combined for persons of white and black† race.

*DENV, dengue virus. †The powerful DENV disease resistance gene(s) in black sub-Saharan Africans might also protect against Zika virus diseases.

In the case of DENV, the ADE phenomenon requires infection of myeloid cells by infectious immune complexes; therefore, it will important to determine if myeloid cells are major targets of Zika virus syndromes in humans. DENV and Zika virus infections are each expressed clinically as febrile exanthema. In monkey and human DENV infections, infected peripheral blood leukocytes circulate transiently just before the appearance of virus in the skin (monkeys) or the appearance of a generalized body rash (humans) (*33*,*46*). In children in Nicaragua, DENV-infected peripheral blood leukocytes circulating late in the viremic period were definitively identified as monocytes (*33*). This finding might be a general phenomenon in viral exanthmata given that infected monocytes also circulate in blood during the rash stage in children with measles (*47*,*48*). A plausible hypothesis is that maculopapular rashes derive from DENV-infected monocytes originating in the bone marrow that are targeted to skin. By analogy, human Zika virus pathogenesis might involve myeloid cells and blood monocytes as infection targets. A search to prove or disprove this hypothesis should be undertaken promptly.

Should Zika virus infect circulating monocytes, this still leaves unanswered the question whether tissue macrophages are an important component of so-called “normal” Zika virus human infections. In A129 mice lacking type 1 interferon receptors, peripheral inoculation of Zika virus produced observable illness and high titers of virus in spleen, liver, and brain, but no attempt was made to identify target cells (*49*). As suggested by excretion of Zika virus in seminal fluid, urine, saliva, and tears, Zika virus might predominantly infect nonmyeloid tissues. As reviewed briefly here, there is ample evidence from studies on human uterine contents, plus validation in mouse models, that Zika virus infects neuronal tissues, a wide range of fetal organs, placental cells, endothelial cells, and reproductive organs. Clearly, we have much to learn about the pathogenesis and pathology of human Zika virus infections. The issue of ADE poses a unique challenge to researchers, the resolution of which rests in the first instance on epidemiologic evidence.
